# Maxingshigan decoction for treating COVID-19: A protocol for systematic review and meta analysis

**DOI:** 10.1097/MD.0000000000023224

**Published:** 2020-11-25

**Authors:** Guoliang Shao, Shaolei Huang, Yuying Cui, Dianhui Yang

**Affiliations:** a Acupuncture and Tuina College, Shandong University of Traditional Chinese Medicine; b Master of Clinical Integration in Traditional Chinese and Western Medicine, College of Traditional Chinese Medicine, Shandong University of Traditional Chinese Medicine; c Department of Acupuncture, Affiliated Hospital of Shandong University of Traditional Chinese Medicine, Jinan, China.

**Keywords:** COVID-19, maxingshigan decoction, meta-analysis, protocol, systematic review

## Abstract

**Background:**

Coronavirus disease 2019 (COVID-19) is a rapidly spreading disease that has been in a public health emergency of international concern since its outbreak in 2020. Due to the complex pathogenesis and susceptibility of COVID-19, many commonly used drugs for the treatment of COVID-19 have not shown excellent clinical effects. Traditional Chinese medicine has a long clinical history of preventing and treating this respiratory infectious disease. Maxingshigan Decoction (MXSG) is widely used in China to treat COVID-19. However, there is no comprehensive and systematic evidence on the effectiveness and safety of Maxingshigan Decoction.

**Methods:**

PubMed, EMBASE, Clinical Trials, the Cochrane Library, Sino Med, and China National Knowledge Infrastructure up to September 2020. This study only screens clinical randomized controlled trials on MXSG for COVID-19 to evaluate its efficacy and safety. Data were extracted by 1 investigator and checked by an independent investigator. Review Manager 5.3 software was used for the data analysis. The dichotomous data is represented by relative risk, and the continuous is expressed by mean difference or standard mean difference, eventually the data is synthesized using a fixed effect model or a random effect model depending on whether or not heterogeneity exists.

**Results:**

The time from a positive diagnosis to a negative result of 2 consecutive nucleic acid tests (not on the same day), cure rate. The results of our research will be published in a peer-reviewed journal.

**Conclusion:**

The purpose of this systematic review is to provide new evidence for the effectiveness and safety of Maxingshigan decoction in the treatment of COVID-19.

**PROSPERO registration number:**

CRD42020211962.

## 1 Introduction

On December 1, 2019, the first case of Coronavirus Disease 2019 (COVID-19) was confirmed in Wuhan, Hubei Province, China, and COVID-19 has spread rapidly around the world and become a serious public health event endangering human life.^[1–3]^ On March 11, 2020, the World Health Organization (WHO) declared COVID-19 a pandemic (WHO Declares global emergency as Wuhan coronavirus spreads). This disease is characterized by being highly contagious, and people of all ages are susceptible to infection (WHO). As of September 30, 2020, more than 30,000,000 confirmed COVID-19 cases have been reported to the World Health Organization, including more than 1,000,000 deaths. According to the conclusion drawn by the WHO, there is no effective way to control the COVID-19.^[4]^ According to clinical experience, antiviral drugs and symptomatic and supportive treatment are often used. However, its therapeutic effect still needs further evaluation.^[5,6]^ Therefore, there is an urgent need to find new treatment methods and measures to prevent disease progression and prevalence.^[7]^ However, traditional Chinese medicine and Chinese herbal medicine have accumulated rich clinical experience and effective formulas in the prevention and treatment of SARS in 2003 and other epidemics (COVID-19).^[8,9]^

As the country with the most experience in treating COVID-19 in China, Maxingshigan Decoction has been widely used in confirmed patients.^[10]^ Maxing-shigan Decoction is composed of ephedra, almonds, gypsum, and licorice. It has multiple functions of antiviral, anti-inflammatory, immune regulation, and fever reduction.^[11]^ Animal pharmacological experiments confirmed that the thrombin and Toll-like receptor signaling pathway were suggested to be essential pathways for Maxingshigan Decoction (MXSG) mediated anti-inflammatory effects.^[8]^ Clinical studies have shown that Maxing Shigan Decoction has a very good effect on the treatment of viral influenza.^[12]^ Therefore, we aim to collect all randomized controlled trials on MXSG Decoction in the treatment of COVID-19, and conduct meta-analysis and systematic reviews to provide evidence-based medicine for the treatment of COVID-19.

## 2 Methods

### 2.1 Protocol registration

This systematic review protocol has been registered in the PROSPERO network (No. CRD42020211962). We used the Preferred Reporting Items for Systematic Reviews and Meta-Analyses Statement^[13]^ guidelines in this study. As this was a review of published literature, ethics committee approval and patient consent were not required. We will update our protocol for any changes in the entire research process if needed.

### 2.2 Inclusion criteria

#### 2.2.1 Study design

The study only selected clinical randomized controlled trials of MXSG for COVID-19 published in both Chinese and English. However, animal experiments, reviews, case reports, and nonrandomized controlled trials are excluded.

#### 2.2.2 Participants

This study included patients who had been clearly diagnosed with the new coronavirus disease. Except that participants must be over 18 years old, there were no strict restrictions on gender and severity of the disease.

#### 2.2.3 Intervention

The test group uses MXSG. The control group can be treated with other treatments except MXSG. There are no obvious restrictions on the dosage of therapeutic drugs and specific intervention routes.

#### 2.2.4 Outcomes

The primary outcomes include the time from a positive diagnosis to a negative result of 2 consecutive nucleic acid tests (not on the same day), total clinical effective rate, improvement rate of lung computed tomography, adverse events. Secondary outcomes are mainly composed of effective rate of clinical symptoms treatment time.

### 2.3 Search methods

The following electronic databases will be searched from December 2019 to December 2020: MEDLINE, PubMed, EMBASE, Web of Science, China National Knowledge Infrastructure, Wan-fang database, Chinese Scientific Journal Database, Chinese Biomedical Literature Databases, and other databases. All published randomized controlled trials (RCTs) about this topic will be included. Exemplary search strategy of PubMed is listed in Table 1. According to the difference of databases, keywords may combine with free words and comprehensive search will be performed.

**Table 1:** PUBMED search strategy.

Number	Search terms
#1	“randomized controlled trial” [Publication Type] OR “controlled clinical trial” [Publication Type] OR “Single-Blind
	Method” [Text Word] OR “random allocation” [Text Word] OR “allocation” [Text Word] OR “RCT” [Text Word]
	OR “RCTs” [Text Word]
#2	“Maxingshigan tang” [All Fields] OR “Maxing shigan tang” [All Fields] OR “Maxingshigan Decoction” [All Fields]
	OR “Maxing shigan Decoction” [All Fields] OR “MXSG” [All Fields] OR “MXSG Decoction” [All Fields]
#3	“COVID-19” [Title/Abstract] OR “COVID19” [Title/Abstract] OR “coronavirus disease 2019” [Title/Abstract]
	OR “coronavirus disease-19” [Title/Abstract] OR“2019 novel coronavirus disease” [Title/Abstract] OR “2019-nCoV disease” [Title/Abstract] OR “2019-nCoV infection” [Title/Abstract] OR “2019-nCoV ” [Title/Abstract] OR “SARSCov2 ” [Title/Abstract] OR “SARS-CoV ” [Title/Abstract] OR “2019 coronavirus ” [Title/Abstract] OR “2019 novel coronavirus infection” [Title/Abstract]
#4	#1 AND #2 AND #3

### 2.4 Data collection and analysis

#### 2.4.1 Selection of studies

**Figure 1:**
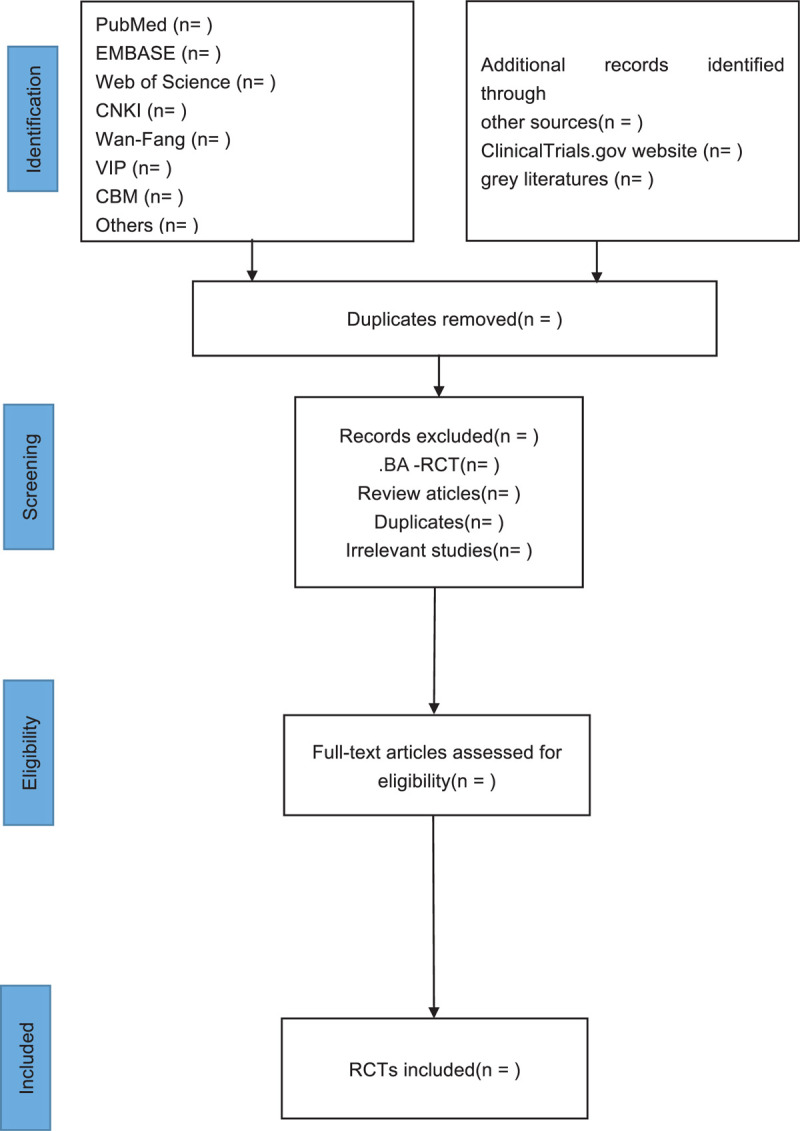
Study flowchart of selected articles for final analysis.

Import all literatures that meet the requirements into Endnote X9 software. Two reviewers (GS and SH) will independently select the studies. They will check the results with each other. When disagreements occur, a third reviewer (DY) will make the final decision. They will read the full texts of all included studies if necessary. Screening operation will flow the diagram of Figure 1. If the full literatures are unable to be obtained or related data are incomplete, we will contact the corresponding author.

#### 2.4.2 Data extraction and management

According to the characteristics of the study, we prepare an excel form for data collection before data extraction. Outcome indicators for eligible studies were independently extracted and filled in the data extraction form by 2 reviewers. The main data extracted are as follows: title, author, year, fund source, sample size, age, sex, duration of disease, interventions, outcome measures, adverse reactions, etc. If there is something unclear, you cannot hesitate to contact authors for more detailed information. The above information was finally cross-checked by 2 reviewers.

#### 2.4.3 Assessment of risk of bias in included studies

The quality assessment of RCTs adopts the risk of bias assessment tool provided by the Cochrane Handbook. The following 7 items, such as random sequence generation, allocation concealment, blinding of participants and personnel, blinding of outcome assessment, incomplete outcome data, selective outcome reporting, and other bias, are evaluated by 3 grades of “low bias,” “high bias,” and “unclear bias.” The discrepancies will get a consistent conclusion by discussing between both reviewers or seeking the third-party consultation.

#### 2.4.4 Data analysis

Different evaluation methods are selected according to the different efficacy indicators. For the dichotomous data, we will choose the effect scale indicator relative risk (RR) with 95% confidence interval (CI) to represent. While the continuous data is expressed as mean difference (MD) or standardized mean difference (SMD) with 95% CI depending on whether the measurement scale is consistent or not. Review Manager software version 5.3 provided by the Cochrane Collaboration will be performed for data synthesis and analysis. The dichotomous data is represented by RR, continuous data is expressed by MD or SMD. The heterogeneity of studies will be evaluated by Q-test and I2statistic with RevMan5.3. The heterogeneity will be deemed as low (I2 < 50%), moderate (50%–75%), and high (I2 > 75%). If there is no heterogeneity (I2 < 50%, *P* > .1), the data are synthesized using a fixed effect model. Otherwise (I2≥50%, *P* < .1), a random effect model is used to analyze. Then subgroup analysis will be conducted based on the different causes of heterogeneity. If a meta-analysis cannot be performed, it will be replaced by a general descriptive analysis.

## 3 Discussion

This is the first study to evaluate the clinical effect and safety of Maxing Shigan Decoction on patients infected with COVID-19. With the advent of winter in the northern hemisphere, many experts predict that the epidemic caused by COVID-19 will once again erupt globally.^[14–16]^ Chinese herbal medicine has unique advantages in the treatment of acute infectious diseases, such as malaria and SARS.^[17]^ During the COVID-19 epidemic, MXSG Decoction played a significant role in improving lung function. This study aims to comprehensively evaluate the effectiveness and safety of MXSG Decoction in the treatment of COVID-19, and to provide more methods for the treatment of COVID-19.

## Author contributions

**Conceptualization:** Dianhui Yang.

**Data curation:** Guoliang Shao, Yuying Cui.

**Formal analysis:** Guoliang Shao, Yuying Cui.

**Funding acquisition:** Dianhui Yang.

**Investigation:** Guoliang Shao, Yuying Cui.

**Methodology:** Guoliang Shao, Yuying Cui.

**Project administration:** Dianhui Yang.

**Resources:** Yuying Cui.

**Software:** Guoliang Shao, Shaolei Huang, Yuying Cui.

**Supervision:** Shaolei Huang, Yuying Cui.

**Validation:** Shaolei Huang.

**Visualization:** Shaolei Huang.

**Writing – original draft:** Guoliang Shao, Yuying Cui.

**Writing – review & editing:** Guoliang Shao, Shaolei Huang.
